# Intraluminal extension of papillary thyroid carcinoma into the Internal Jugular Vein; a case report

**DOI:** 10.1186/s12902-024-01580-x

**Published:** 2024-04-23

**Authors:** Firouze Jafari, Ali Taheri Akerdi, Hossein Abdolrahimzadeh Fard, Mehrdad karajizadeh, Shahram Paydar

**Affiliations:** 1 Department of Pathology, School of Medicine, Shiraz University of Medical Sciences, Shiraz, Iran; 2 Trauma Research Center, Rajaee (Emtiaz) Trauma Hospital, Shiraz University of Medical Sciences, Shiraz, Iran

**Keywords:** Papillary thyroid carcinoma, Thyroidectomy, Intravascular

## Abstract

**Background:**

Papillary thyroid carcinoma (PTC), being the most common thyroid malignancy, is a slow-growing tumor and is usually limited to the thyroid. Extra thyroid extension is uncommon; besides, invasion to the vasculature seems to be extremely rare and usually indicates aggressive nature of the disease.

**Case presentation:**

We present a case of a 40-year-old lady who referred with a palpable neck mass a month after total thyroidectomy which its histopathologic examination revealed follicular variant of PTC; the same variant as prior thyroidectomy. Preoperative ultrasonography failed to comment on the intravascular component of the mass. Surgical procedure confirmed a mass attaching and infiltrating to the internal jugular vein, which turned out to be persistent disease.

**Conclusions:**

Awareness of this entity is important for surgeons, oncologists and radiologist as it can influence patient management.

## Background

The incidence of differentiated thyroid carcinoma is increasing in the United State, which is attributed to early detection of stage I tumors [1]. Papillary thyroid carcinoma (PTC) is the most common thyroid malignancy and accounts for approximately 80% of all thyroid malignancies [2]. Papillary carcinoma remains confined to the thyroid for a long time. Extrathyroid extension into the soft tissues of the neck is found in about one-fourth of cases and adversely affects the prognosis in a very significant fashion [3]. Although involvement of cervical lymph nodes is very common [4], vascular invasion and distant metastasis are not characteristics of PTCs and cases associated with direct spread of the primary tumor or the regional lymph node recurrence to great veins are extremely rare, in particular [5]. We present a case of 40-year-old lady with intraluminal invasion and propagation of Internal Jugular Vein (IJV) with lymph node recurrence of PTC.

## Case presentation

A 40-year-old woman was referred to our center with progressive painless enlargement of right lateral neck mass and a month after total thyroidectomy. She has had history of hypothyroidism for about 20 years. She was on daily levothyroxine. One year before the first surgery, diagnostic fine needle aspiration was done for her, which was not satisfactory for evaluation due to low cellularity. Her concurrent thyroglobulin(TG) and anti-thyroglobulin antibody levels were within normal range. After a while, she underwent ultrasonographic study of neck which showed right thyroid lobe enlargement with heterogenous echopattern containing a few relatively well-defined hypo/iso and hyperechoic nodules accompanied by macrocalcification; the left lobe size and echopattern were normal. Based on this report and the patient desire, thyroidectomy was done for her with preoperative diagnosis of multinodular goiter without any preceding FNA. The surgical pathology report revealed “papillary thyroid carcinoma, follicular variant” in the right lobe, measuring 3 × 2 × 1.5 cm. Tumor multicentricity and lymphovascular invasion were present. No lymph node was submitted.

The patient was referred to the specialist for radioiodine therapy, where the clinician found a 40 × 25 mm mass at the right side of the neck and persistent disease was diagnosed for her. No neck tenderness or dilated veins could be identified. The therapist refused to initiate I-131 treatment and asked for a new ultrasonography.

Subsequent ultrasonography (US) showed a pathologic lymph node at the right side of the neck extending from level 2 to level 4 and also level 6 that encased anterior and anterolateral wall of the right internal jugular vein and also the right common carotid artery. The radiologist was not able to comment on any intravascular masses. The values of TG or antithyroglobulin antibody were not requested. Fine-needle aspiration cytology (FNAC) of the mass confirmed papillary thyroid cells in the enlarged lymph node.

**Fig. 1:**
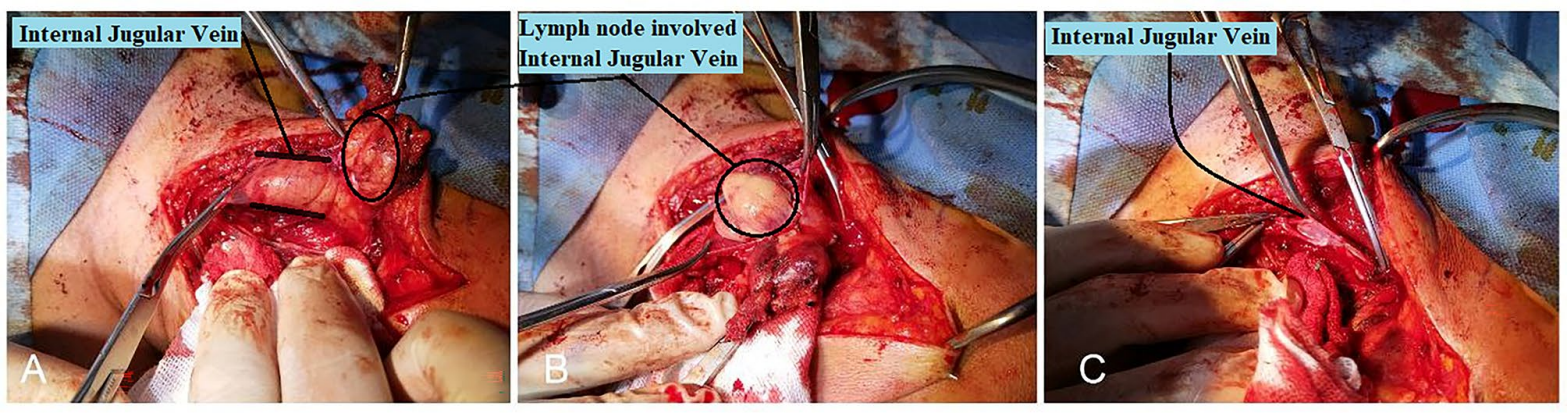
Dumbbelled-shape lymph node, attaching to and infiltrating the IJV. **B** & **C**. Longitudinal venotomy and intraluminal part of the mass which had not tumor thrombus or any endothelial adhesion

Surgery was considered for treatment and in right neck modified radical dissection a 50 × 35 mm dumbbelled-shape lymph node at the lateral side of the common carotid artery was found attaching to and infiltrating the IJV. Longitudinal venotomy revealed the intraluminal part of the mass which had not tumor thrombus or any endothelial adhesion and was easily removed (Fig. 1). In addition, about 18 lymph nodes were isolated from the lateral compartment, one was involved by metastatic carcinoma. Multiple matted lymph nodes were also isolated from the medial compartment, number was not specified, all of which were involved.

All the methods were performed in accordance with relevant guidelines and regulations.

**Fig. 2:**
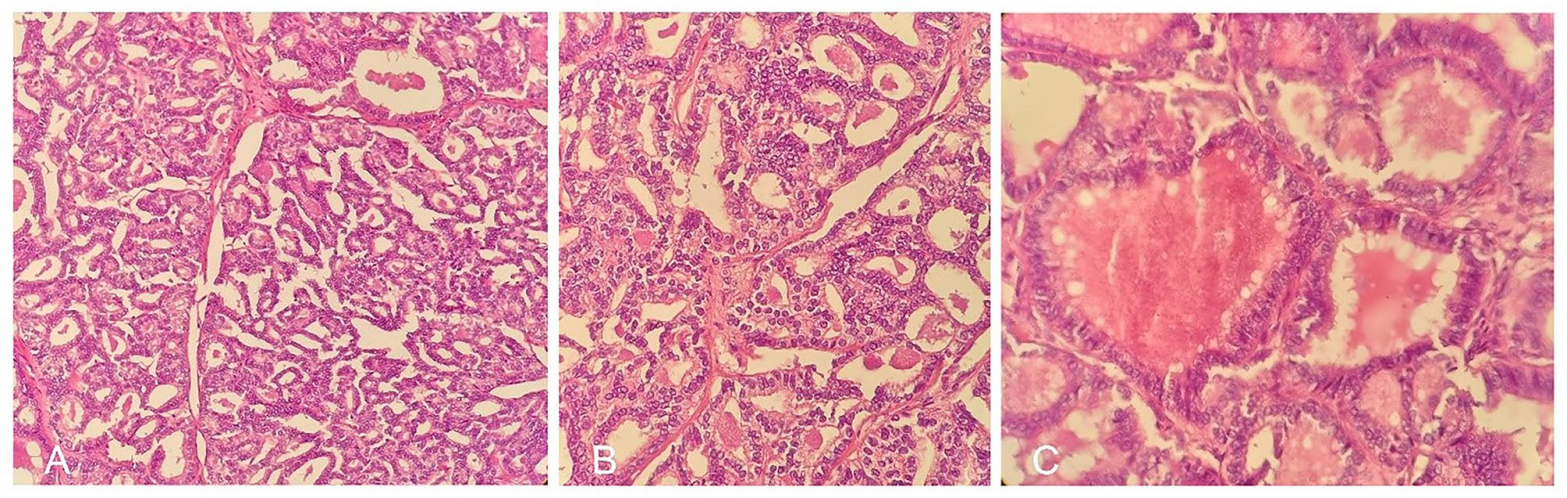
Histopathologic sections of intraluminal mass of IJV. The tumor cells proliferate, forming well-developed follicles. PTC type nuclei are evident. (H & E, original magnifications x10, x20, and x40, respectively)

Histopathology analysis confirmed lymph node involvement by PTC (Fig. 2). The surgery was followed by ablative radioiodine. Whole body iodine scan before I-131 treatment and 2 months after the second surgery revealed significant post-surgical remnant thyroid tissue in the thyroid bed along with upper cervical and upper mediastinal lymph node involvement. Concurrent TG level was more than 100 ng/ml and antithyroglobulin antibody level was 25 IU/ml.

The same study at her last follow-up, about a year after the second surgery, shows radioiodine-avid lesions in the right side of the neck in favor of metastatic lymph node involvement. Her TG level is 76 and antithyroglobulin antibody is 3.6 IU/ml. New course of radiotherapy is considered for her. No sign of distance metastasis is detected.

Written informed consent was obtained from the patient for publication of this case report.

## Discussion and conclusions

The papillary thyroid carcinoma, being the most common subtype of thyroid cancer, is generally a slow-growing tumor and is usually confined to the thyroid gland for a long time [6]. Yet it spreads to the cervical lymph nodes in approximately 50% of the patients and distance metastasis to the lung and/or bone is relatively frequent at the time of diagnosis [5]. Extensive lymph-vascular invasion could be a warning sign of distance metastasis or early relapse. Vascular spread via direct intravascular extension, which may be detected along with the thyroid tumor at the initial presentation or with recurrence in the thyroid bed or more often from a node metastasis, is extremely rare with potential serious complications such as the risk of sudden death from airway compression, tumor embolism or fatal right atrial obstruction [7].

Often the intravascular tumor mass is not clinically detected by examinations alone and diagnosis is made by imaging or intraoperatively. Indeed, ultrasound neck assessment using Valsalva maneuver is advocated as the first investigation to be done when a vein invasion is suspected [6]. Color Doppler or contrast enhanced CT are reliable and accurate to diagnose great cervical vein invasion and plan surgical resection of the tumor [5]. In addition to imaging FNAC is mandatory to rule out poorly differentiated subtypes such as anaplastic carcinoma or a more prevalent entity, metastatic tumors where the prognosis is dismal [6].

Total thyroidectomy, neck dissection and resection of any involved organ accompanied by adjuvant therapy is considered the treatment of choice in locally advanced well-differentiated tumors. Intraluminal extension is not a contraindication for aggressive surgical treatment in differentiated thyroid cancers due to the relatively good prognosis of PTC [8, 9]. In our case the tumor was not attached to the vascular wall and was surrounded by a fibrous capsule which made its removal and vascular repair feasible. However, radical en bloc resection and reconstruction using saphenous vein graft might be indicated in cases where the vascular structures are totally destroyed which may improve survival.

Postoperative high dose radioiodine therapy is administered for all patients in order to eliminate any microscopic residue; and if R2 surgery is done for the patient, whom with gross residual disease irrespective of the histopathologic margin, external beam radiation should also be added to guarantee long term survival [1]. In addition, the patient must be kept on high dose thyroid hormone replacement after completion of treatment [6&8].

In patients with operatively resectable disease, older age, tumor size > 4 cm, R2 resection, and the presence of distant metastases are proven to be predictive of worse disease-specific survival [1].

In summary, vascular invasion with intraluminal propagation is a rare but significant condition in thyroid cancer especially in PTC. Ultrasound imaging of any clinically suspicious neck mass is recommended as aggressive surgical treatment with vascular repair is possible to minimize the risk of potentially fatal complications of the intraluminal masses [8].

## Data Availability

The detail clinic and image data were present in study.

## Abbreviations

PTC: Papillary thyroid carcinoma
IJV: Internal Jugular Vein
US: Ultrasonography
FNAC: Fine-needle aspiration cytology
